# Science under threat around the world

**DOI:** 10.7554/eLife.111487

**Published:** 2026-05-12

**Authors:** Humberto J Debat

**Affiliations:** 1 Instituto Nacional de Tecnología Agropecuaria (INTA) Córdoba Argentina; 2 Department of Molecular Genetics, University of Toronto Toronto Canada

**Keywords:** point of view, science funding, careers in science, early-career researchers, basic research, science, politics, None

## Abstract

Politicians are reducing public funding for science and dismantling scientific institutions for ideological reasons in Argentina and the United States. It appeared as if something similar could happen in the Netherlands, but the collapse of a coalition government led to a reprieve. How should the scientific community respond to such crises?

## Introduction

Much of the current debate about threats to publicly funded science has centered on the United States. But from the perspective of a scientist working in the Global South, what is happening in the US looks less like an anomaly and more like another addition to a growing list of countries where the scientific community is under siege.

In Argentina, where I have a laboratory, the assault on science began in December 2023, when Javier Milei was sworn in as president, and the situation has become worse than most observers thought possible (De Ambrosio and Koop, 2024; Orfila, 2024; Packer, 2026). Elsewhere, researchers have been watching similar dynamics unfold, with the same rhetorical strategies, the same institutional targets, and the same generational consequences. The question facing the global scientific community is whether these disruptions will continue to be treated as separate national problems with separate national solutions, or whether they will be recognized for what they are: a structural threat to the international capacity for scientific enquiry.

The political contexts differ, but the assaults on science follow a recognizable pattern. A government takes office committed to reducing state functions. Science ministries are dissolved or downgraded. Research funding bodies face cuts that exceed what institutions can absorb through reorganization. Specific research areas are cancelled, typically those addressing climate, public health, or social equity. Researchers are characterized in public discourse as unproductive or ideologically captured, and early-career scientists – being the most dependent on public funding, and also the most mobile – begin to leave.

In Argentina, the country’s scientific infrastructure was dismantled so quickly that in March 2024 – three months after Milei became president – 68 Nobel laureates wrote an open letter to him to warn that science and technology in the country were approaching a “dangerous precipice”. Since then science funding has fallen to 0.156% of GDP, its lowest since the 2002 economic crisis, and last December the Agency for Science, Technology and Innovation cancelled previously awarded grants, and stopped making new awards, which means that Argentina is the only country in Latin America that will not allocate public funds to new research projects this year. CONICET, the main research council in Argentina, has also made almost 600 staff scientists redundant (Orfila, 2024), and fellowships offered to 850 early-career researchers by the previous government have been withdrawn (Packer, 2026).

Moreover, political appointees now have the power to close research programmes not aligned with a narrow strategic plan limited to agriculture, energy, mining, health, and the knowledge economy (Gentil, 2025). The effect of this is to redirect what remains of public funding for science in Argentina toward extractive industries, while eliminating research on environmental sustainability, social development, and basic science. This goes beyond a budget reduction and redefines what counts as legitimate scientific enquiry, enforced through administrative authority.

In the United States, more than 7800 research grants already awarded by the National Institutes of Health (NIH) and the National Science Foundation were terminated or frozen by the Trump administration, with a focus on grants for research into misinformation, vaccine hesitancy, infectious disease, and underrepresented groups and minorities (Kozlov et al., 2026). An analysis of more than 2200 terminated NIH grants found that women and early-career researchers were disproportionately affected by these grant cancelations (Oza, 2026). The Trump administration has also launched federal lawsuits against a number of universities in the US. Moreover, more than 25,000 employees have lost their jobs at various science agencies, and the US has withdrawn from international bodies such as the World Health Organization and the Intergovernmental Panel on Climate Change. One consequence of all this has been a large increase in the number of US-based researchers looking to move to Europe (ERC, 2025; Pilkington, 2026; Portala, 2025; Gaind et al., 2025).

In the Netherlands, a far-right coalition implemented cuts exceeding €1bn to higher education and research, eliminating starter grants that supported roughly 1,200 researcher positions (Enserink, 2024; van der Gaag, 2025). In the United Kingdom, three major research councils suspended core funding opportunities in early 2026, and the Science and Technology Facilities Council faces a £162 million shortfall that could force cuts of up to 60% to programs in particle physics, nuclear physics and astronomy (Grove, 2026; Rathbone, 2026).

The multinational character of these cuts matters most for fields where governments provide the most or all of the funding for research. Work on neglected tropical diseases – diseases that disproportionately affect low-income populations – has historically depended on publicly funded institutions in middle-income countries, the very institutions now being dismantled (Barberia and Geffner, 2024). Agricultural research that serves food security rather than commercial profitability depends on agencies like INTA in Argentina and the Department of Agriculture (USDA) in the US, but both are experiencing budget cuts: INTA’s budget has been cut by 40% in two years, and USDA is facing a proposed cut of 20%.

The political drivers in Argentina, the US, the Netherlands and the UK are distinct. The cuts in Argentina are ideological, rooted in a libertarian project that treats public research as a market distortion. The cuts in the US are partly ideological and partly punitive, targeting research the administration considers to be politically objectionable. The cuts in the Netherlands emerged from coalition negotiations where research funding served as a bargaining chip, while the reasons for the cuts in the UK are disputed. However, the outcomes are the same: fewer grants, fewer researchers, fewer functioning laboratories, and a generation of scientists reconsidering whether a research career remains viable. Moreover, certain types of long-term research that cannot be paused – such as ecological monitoring and clinical trials – will suffer permanent data loss, and international collaborations will become harder to establish and sustain.

## What would an adequate response require?

In the past, when one country cut its funding for research, the international system was able to absorb the shock. Scientists relocated, collaborations redistributed effort, and knowledge continued to circulate. However, this ‘solution’ breaks down when multiple countries decide to contract their science base at the same time. Moreover, while emergency fellowships and recruitment programs can rescue individual researchers, they don’t address the destruction of the institutions those researchers once worked in.

An adequate response to the current situation would need to operate at three levels. First, international scientific organizations and funding bodies need mechanisms for making a rapid collective response when a member state dismantles its research infrastructure. The UNESCO Recommendation on Science and Scientific Researchers, ratified by 195 member states, already establishes norms for scientific freedom and institutional autonomy, with a four-yearly monitoring cycle (UNESCO, 2017), but this lacks enforcement mechanisms and its reports have had limited visibility.

The International Science Council, through its Committee for Freedom and Responsibility in Science, and national academies coordinating through the InterAcademy Partnership, could strengthen this framework by establishing formal early-warning protocols for institutional threats to science. This work could be done in collaboration with bodies working to protect democratic institutions more broadly, such as the International Institute for Democracy and Electoral Assistance and the V-DEM Project, and with campaign groups such as Scholars at Risk. One goal would be to develop multilateral norms that codify the independence of research councils and science ministries, making their dissolution a matter of international diplomatic concern rather than a purely internal budget decision.

The value of these efforts does not depend on whether the governments in question comply. Formal monitoring creates a documented record that supports domestic legal challenges, informs diplomatic pressure from partner nations, and provides the evidentiary foundation for rebuilding science systems after political transitions, as demonstrated in Brazil, where international documentation of the damage inflicted on science under the Bolsonaro administration informed the post-2023 reconstruction of the country’s research funding and institutional framework (Crestana et al., 2023).

Second, countries and international organizations still investing in research need to plan strategically for absorptive capacity. Emergency fellowships, such as those offered by the European Research Council and the European Union, address the displacement of individual scientists, but there is also a need for sustained funding to accommodate displaced research teams and their work, not merely individual researchers. Concretely, this means funding agencies in stable countries should create dedicated program lines that allow incoming researchers to continue multi-year projects, including the transfer of datasets, biological collections, and field monitoring programs, rather than requiring them to start entirely new work. Such investment should be treated as a strategic contribution to maintaining global research capacity from which the host country also benefits.

Third, the scientific community must confront the political conditions that enable these attacks. The rhetoric used to attack publicly-funded science – describing researchers as unproductive, dismissing their work as frivolous, labelling universities and scientific institutions as bloated – follows a recognizable pattern across countries and political movements. Countering this requires that scientific societies and professional organizations – both national and international, both discipline-specific and multidisciplinary societies – invest systematically in building alliances with the constituencies that depend on publicly generated knowledge: patient advocacy groups, agricultural producer associations, communities exposed to environmental degradation, and industries built on foundational research. These alliances must be constructed during periods of stability, not improvised during crises. In Argentina, for example, researchers at INTA (where I work) have longstanding relationships with farming communities who depend on publicly developed crop varieties and extension services, the kind of constituency whose political voice carries weight that scientific arguments alone often do not.

## Outlook

Although the courts have reversed some of the cuts announced by the Trump administration in the US, and the coalition government in the Netherlands collapsed in June 2025, a great deal of damage has already been done (and the situation in Argentina remains dire). Early-career researchers have left science, never to return. Laboratories have closed and will not reopen. Until now the scientific community has responded to each assault on science in a particular country in isolation, but this approach is no longer adequate. The complexity of modern research means that international coordination and collaboration have become essential prerequisites for tackling the biggest challenges in science. We now have to use the same approach to defend science.
